# Microsatellite Instability in Sarcoma: Fact or Fiction?

**DOI:** 10.5402/2012/473146

**Published:** 2012-07-18

**Authors:** Michael J. Monument, Stephen L. Lessnick, Joshua D. Schiffman, Rl. Tx. Randall

**Affiliations:** ^1^Sarcoma Services, Department of Orthopaedics, Huntsman Cancer Institute, University of Utah School of Medicine, 2000 Circle of Hope, Salt Lake City, UT 84112, USA; ^2^Department of Oncological Sciences, Huntsman Cancer Institute, University of Utah School of Medicine, 2000 Circle of Hope, Salt Lake City, UT 84112, USA; ^3^Division of Pediatric Hematology/Oncology, Huntsman Cancer Institute, University of Utah School of Medicine, 2000 Circle of Hope, Salt Lake City, UT 84112, USA; ^4^Center for Children's Cancer Research (C3R), Huntsman Cancer Institute, University of Utah School of Medicine, 2000 Circle of Hope, Salt Lake City, UT 84112, USA

## Abstract

Microsatellite instability (MSI) is a unique molecular abnormality, indicative of a deficient DNA mismatch repair (MMR) system. Described and characterized in the colorectal cancer literature, the MSI-positive phenotype is predictive of disease susceptibility, pathogenesis, and prognosis. The clinical relevance of MSI in colorectal cancer has inspired similar inquisition within the sarcoma literature, although unfortunately, with very heterogeneous results. Evolving detection techniques, ill-defined sarcoma-specific microsatellite loci and small study numbers have hampered succinct conclusions. The literature does suggest that MSI in sarcoma is observed at a frequency similar to that of sporadic colorectal cancers, although there is little evidence to suggest that MSI-positive tumors share distinct biological attributes. Emerging evidence in Ewing sarcoma has demonstrated an intriguing mechanistic role of microsatellite DNA in the activation of key EWS/FLI-target genes. These findings provide an alternative perspective to the biological implications of microsatellite instability in sarcoma and warrant further investigation using sophisticated detection techniques, sensitive microsatellite loci, and appropriately powered study designs.

## 1. The Essence of Microsatellite DNA

The biological precedence of tandem nucleotide repeats scattered throughout the human genome has intrigued scientific inquiry since these genetic elements were first characterized in the early 1980s. More precisely, the term *microsatellite DNA* refers to tandem iterations of simple sequence motifs dispersed throughout the genome. The majority of microsatellite DNA is comprised of mono-, di-, tri- and tetra-nucleotide repeats, and these repetitive elements constitute ~3% of the human genome [1]. Current estimates suggest that there are approximately one million microsatellite loci within the human genome, and the vast majority of these sequences are situated within noncoding regions such as intronic and intergenic segments. Consequently, microsatellite DNA has been long regarded as “junk DNA” with a poorly understood biological function. The repetitive nature of microsatellite DNA renders it more susceptible to mutagenesis during DNA replication and furthermore, the lack of evolutionary pressure on these noncoding regions has licensed an impressive rate of microsatellite polymorphisms in the human population overtime. Compared to coding regions of the genome, microsatellite loci are genetically diverse, characterized by high heterozygosity indices and numerous alleles for any given loci [2]. The polymorphic nature of microsatellite DNA across the human population implies a high basal spontaneous mutation rate in these sequences, and although the rate of new mutations is increased compared to other genomic sites, the overall frequency of mutations remains quite low, on the order of 5 × 10^−4^ to 5 × 10^−5^ [3]. Most commonly microsatellite replicative errors occur in the form of a length expansion [4]. 

The mechanism by which microsatellite DNA undergoes a length mutation is commonly believed to occur via “replication slippage,” where the replicating DNA strand transiently dissociates from the DNA template and reanneals out of frame in denominations of the repeat motif [5]. In general, the intrinsic constitution of the microsatellite dictates its replicative instability, where mutation frequency is proportional to the overall microsatellite length and inversely proportional to the size of the repeat motif [6]. The molecular checkrein of this erroneous process is mediated by the DNA “mismatch repair” (MMR) system, where postreplication errors are identified and enzymatically corrected, thus maintaining microsatellite stability. In eukaryotic cells, this surveillance and repair process is mediated by two highly conserved protein complexes: MutS (MSH2, MSH3, MSH6) and MutL (MLHI, PMS2), which function in concert to identify and simultaneously correct replicative errors, respectively [7]. Unchecked errors in DNA replication resulting in expansions or contractions of microsatellite loci are known as microsatellite instability (MSI). Typically, the repeat undergoes expansion or contraction in multiples of the repeat motif. For example, MSI involving a trinucleotide repeat will increase or decrease in size by a multiple three base pairs and so forth. 

## 2. Microsatellite Instability in Cancer?

Microsatellite instability was discovered and characterized nearly 20 years ago in patient-derived colorectal tumors [8, 9]. These seminal papers identified a distinct subset of colorectal tumors demonstrating somatic amplifications of various dinucleotide microsatellite loci, and furthermore, this subset of microsatellite unstable tumors was phenotypically distinct, more commonly located in the proximal colon and associated with superior patient survival [8, 9]. Propelling the momentum of this discovery was the observation that microsatellite instability was a characteristic finding of tumors in patients diagnosed with hereditary nonpolyposis colorectal cancer (HNPCC) [10, 11]. HNPCC, also referred to as Lynch syndrome is an autosomal dominant condition, representing 1–3% of colorectal carcinomas. Affected patients harbor germline mutations in the MMR genes, most commonly MSH2 and MLH1, predisposing to the development of colorectal, ovarian, and endometrial tumors at a young age [12]. When germline mutations are present in the MMR genes, the cumulative risk of developing colorectal cancer is 60–70% and 40–80% for endometrial cancer in females [13, 14]. MSI is now recognized as a defining phenotypic feature present in >90% of HNPCC [12, 15]. MSI is less common in sporadic colorectal cancers, observed in only 10–15% of tumors and is attributed to either germline mutations or epigenetic silencing of the MMR genes [16]. Interestingly, MSI-positive sporadic colorectal carcinomas behave similarly to their HNPCC counterparts, demonstrating a defined pattern of tumor anatomy, biological behavior, and a more favorable clinical prognosis [8, 9, 17].

This important molecular signature has lead to the development of standardized screening guidelines, diagnostic protocols and even a reference panel (*The Bethesda Panel*) of mono- and di-nucleotide microsatellite loci sensitive to the development of microsatellite instability [18, 19]. MSI is detected by numerous techniques, which ultimately compares the length microsatellite sequences in tumor and normal cells, usually peripheral leukocytes. MSI-positive tumors are then further subclassified into MSI-low (MSI-L) and MSI-high (MSI-H) phenotypes; MSI-H tumors demonstrate MSI at 2 or more microsatellite loci, and most importantly, this subset of MSI-positive tumors posses the favorable biological attributes ascribed to MSI-positive tumors [18, 19]. These guidelines have since been validated as accurate molecular screening tools for the prediction of germline mutations in the DNA mismatch repair system [15], influencing genetic counseling and surveillance protocols. Additionally, recent systematic reviews and meta-analyses have recapitulated earlier findings of improved survival and a characteristic chemosensitivity profile of MSI-H colorectal carcinomas compared to tumors with an intact DNA mismatch repair system [17, 20–22]. The discovery of MSI in CRC and HNPCC represents a novel discovery linking microsatellite DNA and the MMR system to oncogenesis. MSI is now revered as a distinctive phenotype in cancer cells harboring mutations or epigenetic silencing of the DNA mismatch repair genes and consequently, a valuable predictor of cancer susceptibility and a clinically relevant marker of tumor biology.

## 3. Is MSI Common in Sarcoma and a Potential Molecular Predictor of Disease Behavior?

The determination of MSI and defects of the MMR system in sarcoma has gained increasing attention for several evolving reasons. Firstly, there remains a paucity of clinically relevant and easily assayed molecular biomarkers in sarcoma. The characterization of MSI in colorectal cancer and the informative nature of this mutator phenotype have provided optimism that similar findings may be observed in a distinct subset of sarcomas. Secondly, although microsatellite DNA is primary in noncoding regions of the genome, clusters of microsatellite DNA are found in coding and flanking regions of genes identified in sarcomagenesis. For example, the *TGF*β*RII* gene contains a mononucleotide microsatellite (A)_10_, which is commonly mutated in MSI-positive tumors [23]. *TGF*β*RII* signalling is known to inhibit cell growth and proliferation and therefore is considered to have a tumor-suppressor role in oncogenesis [24, 25]. EWS/FLI-mediated silencing of *TGF*β*IIR* gene expression has been implicated in the process of oncogenic transformation in Ewing sarcoma cells [24, 26, 27]. The proapoptotic Bcl-2 family gene, *BAX*, also harbors a coding microsatellite region [23]. *BAX* is downstream target of p53-mediated apoptosis [28] and dysregulated p53 signalling plays a pivotal role in the oncogenic transformation of various sarcoma types [29–31]. Interestingly, members of the MMR genes, MSH6 and MSH3, also harbor coding microsatellites, which are commonly mutated in MSI-positive tumors [23]. Other sarcoma-relevant genes harboring coding microsatellite regions also include IGF-receptor-II and the histone deacetylase, HDAC2 [32–35]. Finally, recent investigations in Ewing sarcoma have demonstrated a mechanistic role of microsatellite DNA in EWS/FLI-mediated gene activation [36], which hypothetically renders these key target genes extremely biologically sensitive to repeat expansion or contraction.

Shortly after the characterization of MSI in CRC, numerous studies emerged addressing the issue of MSI in sarcoma.  Table 1 summarizes this literature, highlighting the sarcoma types assessed, methods of MSI assessment, frequency of MSI, and any clinical observations associated with the molecular diagnosis of MSI. Not unlike many other sarcoma studies, the infrequent nature of sarcomas has limited the majority of these studies to heterogeneous samplings of various sarcoma subtypes, ubiquitously underpowered to detect meaningful clinical outcomes. Furthermore, the methodology employed in these studies is also quite diverse; with some series utilizing fresh frozen tumor specimens and peripheral blood as control genomic DNA, while others have harvested tumor cells and control cells from formalin-fixed and paraffin-embedded (FFPE) tissue blocks. The microsatellite panels investigated were also very inconsistent; a few studies assessed a consistent panel of microsatellite loci validated for the determination of MSI in colorectal cancer, while many others utilized microsatellite loci flanking chromosomal regions know to house common genes involved in sarcomagenesis, such as the *p53* and *RB* loci on chromosomes 13q and 17p, respectively. Many studies assessing these sarcoma-specific microsatellite loci were often simultaneously assessing MSI and loss of heterozygosity (LOH) as a collective phenotype of allelic imbalance.

Not surprisingly, these studies do not provide conclusive evidence for or against the presence of microsatellite instability in sarcoma. Five studies observed a high frequency of MSI [37–41], ranging from 25–50%, where the 8 remaining studies report a low frequency of MSI [42–49], ranging from 0–14%. Two independent studies came to similar conclusions that the absence of MSI in clear cell sarcoma can be used as a useful adjunct (in addition to detection of the t(12 : 22) translocation) when differentiating clear cell sarcoma from malignant melanoma of soft parts [44, 48]. Three studies that detected MSI also assessed clinical outcomes; 2 of which concluded MSI-positive tumors were predictive of an inferior clinical outcome [38, 40], while the remaining study found no clinical correlate [49]. Importantly, these studies were not methodologically devised to accurately assess clinical parameters as a dependent outcome of microsatellite status with appropriate scientific precision. It also worthy to mention that the MSI-H phenotype overall was infrequently observed across all studies. Of the 10 studies that observed the MSI-positive phenotype, only 4 detected instability at >1 microsatellite loci: Belchis et al. [37], 2/8; Martin et al. [38], 3/7; Klingler et al. [39], 3/6; Ohali et al. [40], 4/11 tumor specimens.

The accurate detection and interpretation of MSI is a complex and persistently evolving subject. Consequently, a variety of methodological issues must be appreciated when interpreting these discordant results. The accuracy of MSI detection is highly dependent on the molecular techniques used for assessment. Firstly, Taq polymerase is prone to slippage errors during PCR amplification. The slippage rate of Taq polymerase increases with an increasing number of repeats and is inversely proportional to the length of the microsatellite repeat unit [50, 51]. The inherent replication slippage of Taq polymerase produces PCR artifacts known as “stutter bands” that can blur the distinction between an in vitro replication error and a true microsatellite allele when using low-resolution gel detection techniques. Taq polymerase also possesses terminal deoxynucleotide transferase (TDT) activity, adding additional nucleotide units to the newly synthesized PCR products [52]. This erroneous feature can be avoided using T4 DNA polymerase [52]. All of the aforementioned studies used Taq polymerase for microsatellite amplifications. Additionally, PCR products resolved and visualized on polyacrylamide and agarose-based gels are susceptible to gel migration errors. This is further confounded using autoradiographic detection systems prone to detection errors, which are often insensitive to low-signal bands and misinterpret the intensity of higher-magnitude signals [50]. These deficiencies limit the detection of more subtle changes in microsatellite length and risk overestimating microsatellite stability. The magnitude of measurable microsatellite instability has been subclassified into type I and type II MSI, where type I MSI is ascribed to “significant alterations” in microsatellite length (>6–8 bp), and type II MSI refers to “minor alterations” in microsatellite length of 2–4 bp [8]. Gel electrophoresis and autoradiographic detection methods are unlikely to detect type II MSI. Interestingly, mice and cell lines deficient in the MMR system predominantly display a Type II MSI phenotype [53, 54].

**Figure 1:**
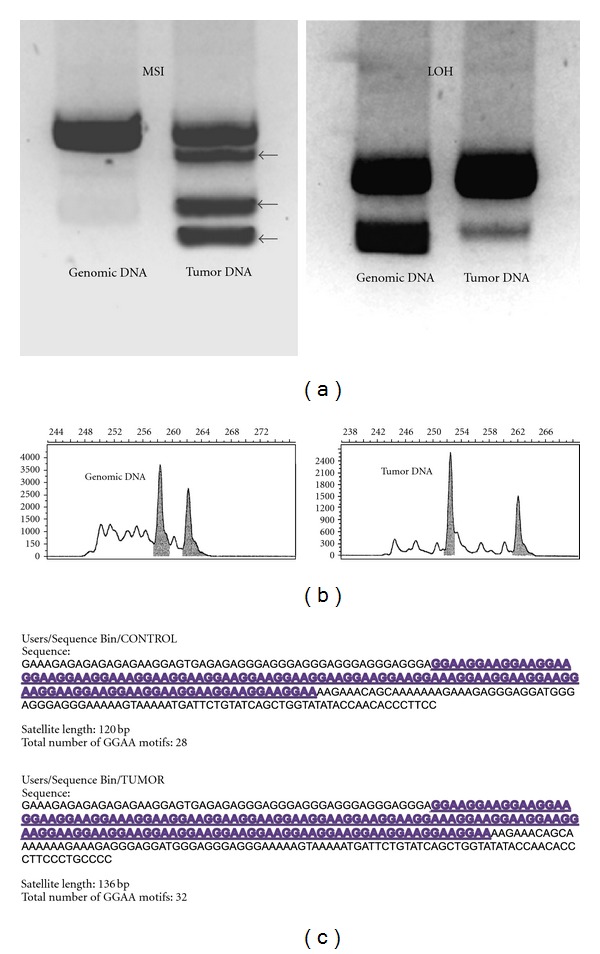
(a) Microsatellite instability was originally assessed using gel electrophoresis and autoradiographic detection. In the left panel, additional bands (black arrows) in the tumor lane illustrate multiple contracted microsatellite alleles relative to the genomic control lane. In the right panel, an information (heterozygous) microsatellite is shown in the genomic control sample, and a significant loss of signal intensity for the smaller allele is observed in the tumor sample, characteristic of allelic imbalance/loss of heterozygosity (LOH). (b) Microsatellite loci are now commonly assessed using fluorescent PCR amplifications, capillary electrophoresis, and automated sequencing techniques. Laser scanners detect fluorescent PCR products and generate a chromatogram displaying microsatellite allele frequencies. Note in the tumor panel, one of the alleles has undergone contraction, depicting MSI in this tumor specimen. (c) Subcloning and direct sequencing of microsatellite amplifications can yield high resolution of the microsatellite sequence and can detect subtle changes in microsatellite constitution. This method of analysis is more time consuming, although programmed bioinformatics software can greatly assist the interpretation of high-volume data. Panel (b) modified with permission from Vilar and Gruber [57].

 The advent of fluorescent-labeled PCR and automated sequencing methods negates many of the shortcomings of traditional electrophoresis and autoradiographic detection systems. Fluorescent-labeled nucleotides are added to the PCR condition and synthesized amplicons from independent samples are coelectrophoresed and quantitatively assessed using a laser scanner [50]. Using fluorescent labels of similar molecular size, coelectrophoresis and a migration standard, migration errors are minimized, and smaller microsatellite alterations can be accurately detected [50, 55]. Six of the studies listed in Table 1 used a version of this newer technique, with 4 of these studies detecting MSI (range: 11%–40% of tumors) [41, 48, 49, 56]. Microsatellite amplifications can also be assessed via subcloning and direct sequencing techniques, yet although this technique has the advantage of being highly sensitive and accurate for detecting small microsatellite polymorphisms, it is a time consuming process and not conducive to high-volume analyses.  Figure 1 illustrates some the different techniques used to detect microsatellite instability.

A very important technical consideration pertains to the microsatellite loci assessed in determining the MSI-positive phenotype. Based on the plethora of literature detailing this topic in colorectal cancer, the ideal microsatellite panel should consist of the following: microsatellite harboring mono- and di-nucleotide repeats, a panel of ≥5 informative loci, microsatellite loci sensitive to mismatch repair deficiency and avoidance of microsatellite loci in regions highly susceptible to copy number deletion (loss of heterozygosity) [18, 19, 58]. Microsatellite analysis is routinely employed in sarcoma research, assessing tumors for global genomic instability, LOH, copy number alterations and has been extremely useful in mapping novel candidate tumor suppressor genes. The Bethesda consensus guidelines advise that in many instances, LOH cannot be accurately delineated from MSI [18, 19]. Numerous studies summarized in Table 1 assessed microsatellite loci susceptible to LOH and made subsequent conclusions of MSI based on these loci [21, 37, 45, 47]. A simple strategy to avoid this confounding issue would be to assess the Bethesda panel of microsatellite loci for MSI separately to any loci assessed for LOH and allelic imbalance. Only three studies listed in Table 1 assessed MSI in strict accordance with the Bethesda guidelines [46, 48, 49], and MSI was infrequently observed these series. Furthermore, none of the three series detected the MSI-H phenotype.

Given the discrepancy of results and the methodological heterogeneity used to assess MSI, it remains difficult to ascertain whether MSI is a prominent molecular phenotype in common sarcomas. The incidence of MSI in sporadic colorectal carcinomas is routinely reported around 15% [8, 17–23, 58] and irrespective of the variability observed in the sarcoma literature, 10/14 studies listed in Table 1 observed MSI in various sarcoma types ranging in frequency from 6%–50%. Certainly the MSI-positive phenotype is not ubiquitous in sarcoma as it is in HNPCC, which is instinctive since germline mutations of the MMR system have not been reported in sarcoma, but instead, it is more likely that MSI is observed with at least the same frequency as observed in sporadic CRC. A very important point to reiterate, however, is that the MSI-H phenotype was observed infrequently across all studies. This is the informative phenotype referenced in the colorectal literature, where MSI-L tumors behave similarly to microsatellite stable tumors [17, 20–22]. 

Optimistically, it was hoped that the identification of the MSI phenotype in sarcoma could similarly predict tumor behavior and clinical outcomes. From the available studies in sarcoma, there is no compelling evidence to suggest a predictive value of microsatellite instability in sarcoma. The clinical conclusions from the sarcoma literature should be interpreted cautiously though as in contrast to the colorectal cancer literature, which is comprised of clinical studies and meta-analysis assessing 100s–1000s of patients [8, 17–21], the sarcoma literature is hindered by small number studies and a low reported frequency of the MSI-H phenotype. Summarizing the information available in Table 1, it does not appear that MSI, especially the predictive MSI-H phenotype is routinely detectable and clinically predictive as compared to the colorectal literature. This maybe a product of small sample numbers, sample heterogeneity, infrequent deficiencies of the MMR system in sarcoma, or simply a lack of consensus regarding the most appropriate sarcoma-specific microsatellite loci to be assessed. If future studies are designed to assess the relevance of MSI instability in sarcoma, larger numbers, modern detection techniques, and an appropriate microsatellite loci panel are essential. 

## 4. Microsatellite Instability in Ewing Sarcoma?

Recent evidence in the Ewing sarcoma literature has demonstrated a novel, mechanistic necessity of microsatellite DNA during EWS/FLI-mediated oncogenesis [36, 59]. Like numerous other sarcomas, Ewing tumors harbor a characteristic somatic translocation. The N-terminal, transcriptional activating domain of the* EWS* gene is fused with the DNA-binding C-terminus of a member of the *ETS* family of transcription factors. The most common translocation, EWS/FLI1 (t22 : 11) is observed in roughly 85%–90% of Ewing tumors [60]. Genomewide microarray and ChIP-chip datasets have identified numerous target genes directly bound and regulated by EWS/FLI [36, 61]. Additionally, these datasets have demonstrated that a subset of these target genes house a GGAA microsatellite response element embedded within the promoter region. EWS/FLI binds with high affinity to these GGAA-rich microsatellites, which are located roughly 1.1–1.6 kb upstream from the transcriptional start site. Two of these highly enriched EWS/FLI-targets, *CAV1* and *NR0B1*, are necessary for oncogenic transformation [62, 63], while a third target, *GSTM4,* is associated with therapeutic resistance and inferior clinical outcomes [64]. The most highly enriched EWS/FLI target gene is *NR0B1*, which contains a 102bp microsatellite, consisting of 25 GGAA motifs and 2 single-base insertions [36]. Characterization of the *NR0B1* promoter has demonstrated that a minimum of four GGAA motifs are needed for EWS/FLI-mediated gene activation, which increases exponentially with an increasing number of GGAA repeats [36, 59]. Length polymorphisms of the *NR0B1 *GGAA microsatellite have also been observed across various Ewing sarcoma cell lines, where a significant correlation between overall microsatellite length and *NR0B1* gene expression is observed [65]. These data represent new evidence supporting a mechanistic role of microsatellite DNA in the EWS/FLI-mediated activation of key determinant genes driving Ewing sarcoma oncogenesis. 

The importance of microsatellite DNA in Ewing sarcoma oncogenesis has stimulated renewed interest in the determination of microsatellite instability in this subtype of sarcoma. If an increasing number of GGAA repeat motifs enhances EWS/FLI binding and gene activation, microsatellite instability within these response elements has potential for significant biological ramifications in Ewing sarcoma cells. Three studies listed in Table 1 investigated MSI specifically in Ewing sarcoma. In the series reported by Ohali et al., MSI instability was observed in 44% (11/23) cases, where MSI-H phenotype and genomic instability at >30% of testable loci were associated with an inferior clinical outcome, although not statistically significant (*P* = 0.13 and *P* = 0.28, resp.). On the contrary, studies by Alldinger et al. [49] and Ebinger et al. [46] documented low rates of MSI in Ewing sarcoma tumors, none of which were the MSI-H phenotype. Arguably, the series by Alldinger et al. utilized the most stringent methodology of all the studies listed in Table 1, assessing 55 Ewing sarcoma samples, testing MSI with sensitive automated sequences techniques and assaying microsatellite loci recommended by the Bethesda guidelines. Furthermore, clinical data as part of two large European clinical trials was available for 49 specimens, and no differences were noted in overall survival when comparing tumors with and without MSI. 

 Despite the discordant data in Ewing sarcoma, the discovery of GGAA microsatellites as EWS/FLI-response elements recapitulates the need for a more detailed assessment of MSI in Ewing sarcoma. Certainly, instability within these GGAA response elements has potential to significantly affect gene expression of key EWS/FLI targets. Given the tetranucleotide composition of the GGAA repeat motif, these microsatellites may theoretically be more intrinsically stable than simple mono- and di-nucleotide repeats. However, the *NR0B1* microsatellite is quite polymorphic across populations, with extremely large repeats, containing as many as 70 GGAA motifs, observed in both African and European subjects (unpublished data). The shear magnitude of these 70 repeat microsatellites would be more intrinsically unstable than the 20–25 GGAA repeats observed in many of the Ewing sarcoma cell lines. 

In summary, microsatellite instability is a captivating oncological observation, highlighting the contribution of microsatellite DNA and mismatch repair system in the process of oncogenesis. Despite regular observations in the colorectal literature, the detection and clinical ramifications of MSI in sarcoma are less consistent. Certainly there is evidence to suggest that many sarcomas display the MSI-positive phenotype although this finding may relate more to generalized genomic instability than a deficient mismatch repair system. The observation of microsatellite response elements in Ewing sarcoma provides an alternative motivation to reassess microsatellite instability in this context. Future efforts in this topic must ensure that studies are appropriately designed; employing advanced sequencing techniques, sensitive microsatellite markers, and adequately powered to accurately measure clinical outcomes.

## Figures and Tables

**Table 1:** Chronological summary of MSI assessment in sarcomas.

Authors	Year	Sarcoma population	Tissue source	Method of microsatellite assessment	Bethesda consensus panel	Frequency of MSI	Clinical correlation
Wooster et al. [42]	1994	Soft-tissue sarcomas (*n* = 18)	Unspecified tumor preparation; peripheral blood (genomic control DNA)	PCR; 6% agarose electrophoresis, subcloning, and sequence analysis	0/5 markers assessed	11% (2/18) MSI at one loci	Not assessed
Belchis et al. [37]	1996	Osteosarcoma (*n* = 18)	FFPE tumor specimens; unspecified normal tissue	PCR; gel electrophoresis; autoradiography	0/5 markers assessed	44% (8/18) with MSI at ≥1 loci (2/8 were MSI-H)	Not assessed
Tarkkanen et al. [43]	1996	Bone sarcomas (*n* = 29)	Unspecified tumor preparation; peripheral blood (genomic control DNA)	PCR; gel electrophoresis; autoradiography	0/5 markers assessed	No MSI observed	n/a
Martin et al. [38]	1998	Bone and soft tissue (*n* = 16)	Fresh frozen tumor and peripheral blood (genomic control DNA)	PCR; gel electrophoresis; autoradiography	0/5 markers assessed	44% (7/16) with MSI at ≥1 loci (3/7 MSI-H)	MSI associated with poor clinical outcome
Aue et al. [44]	1998	Clear cell sarcoma and melanoma (*n* = 11)	FFPE specimens (tumor and genomic control DNA)	PCR; gel electrophoresis; autoradiography	0/5 markers assessed	No MSI observed	Conclude MSI analysis can be used to differentiate CCS from melanoma
Klinger et al. [39]	2000	Chondrosarcoma (*n* = 12)	FFPE specimens (tumor and genomic control DNA)	PCR; gel electrophoresis; autoradiography	0/5 markers assessed	50% (6/12) with MSI at ≥1 loci (3/6 MSI-H)	Not assessed
Entz-Werle et al. [45]	2003	Osteosarcoma (*n* = 54)	Fresh frozen tumor and peripheral blood (genomic control DNA)	Fluorescence-based PCR; automated sequencing	1/5 markers assessed	No MSI instability observed	n/a
Ohali et al. [40]	2004	Ewing sarcoma (*n* = 23)	Fresh frozen tumor and peripheral blood (genomic control DNA)	PCR; gel electrophoresis; autoradiography	2/5 markers assessed	48% (11/23) with MSI at ≥1 loci (4/11 MSI-H)	MSI associated with poor clinical outcome
Rucińska et al. [41]	2005	Soft-tissue sarcomas (*n* = 20)	Fresh frozen tumor and peripheral blood (genomic control DNA)	Fluorescence-based PCR; automated sequencing	1/5 markers assessed	100% (8/8) of high-grade sarcomas with MSI at ≥1 loci (4/8 MSI-H); No MSI in low-grade sarcomas	Not assessed
Ebinger et al. [46]	2005	Ewing sarcoma (*n* = 18)	FFPE specimens (tumor and genomic control DNA)	Unspecified	5/5 markers assessed	6% (1/18) with MSI at one loci	Not assessed
Kawaguchi et al. [56]	2005	STS (*n* = 40)	Fresh frozen tumor and normal tissue	Fluorescence-based PCR; automated sequencing	1/5 markers assessed	25% (10/40) with MSI at ≥1 loci (2/10 MSI-H)	Not assessed
Entz-Werlé et al. [47]	2005	Osteosarcoma (*n* = 68)^∗∗^ same patient cohort as [45]	Fresh frozen tumor and peripheral blood (genomic control DNA)	Fluorescence-based PCR; automated sequencing	1/5 markers assessed	No MSI instability observed	n/a
Garcia et al. [48]	2006	Clear-cell sarcoma (*n* = 9)	FFPE specimens (tumor and genomic control DNA)	Fluorescence-based PCR; automated sequencing	5/5 markers assessed	11% (1/9) with MSI at one loci	Conclude MSI analysis can be used to differentiate CCS from melanoma
Alldinger et al. [49]	2007	Ewing sarcoma (*n* = 55)	FFPE tumor specimens and peripheral blood (genomic control DNA)	Fluorescence-based PCR; automated sequencing	5/5 markers assessed	14% (8/55) with MSI at one loci	MSI not predictive of clinical outcome

MSI: microsatellite instability; STS: soft-tissue sarcoma; FFPE: formalin-fixed, paraffin-embedded; PCR: polymerase chain reaction; CCS: clear-cell sarcoma.
